# Associations between ADC histogram analysis values and tumor-micro milieu in uterine cervical cancer

**DOI:** 10.1186/s40644-024-00814-4

**Published:** 2024-12-20

**Authors:** Alexey Surov, Jan Borggrefe, Anne-Kathrin Höhn, Hans-Jonas Meyer

**Affiliations:** 1 Institute of Radiology, Neuroradiology and Nuclear Medicine, Johannes-Wesling-Klinikum Minden, Ruhr-University Bochum, Hans Nolte Str. 1, 32429 Bochum, Minden, Germany; 2 Institute of Pathology, University of Leipzig, Leipzig, Germany; 3 Diagnostic and Interventional Radiology, University of Leipzig, Leipzig, Germany

**Keywords:** MRI, ADC, Cervical cancer, Tumor-stroma ratio

## Abstract

**Background:**

The complex interactions of the tumor micromilieu may be reflected by diffusion-weighted imaging (DWI) derived from the magnetic resonance imaging (MRI). The present study investigated the association between apparent diffusion coefficient (ADC) values and histopathologic features in uterine cervical cancer.

**Methods:**

In this retrospective study, prebiopsy MRI was used to analyze histogram ADC-parameters. The biopsy specimens were stained for Ki-67, E-cadherin, vimentin and tumor-infiltrating lymphocytes (TIL, all CD45 positive cells). Tumor-stroma ratio (TSR) was calculated on routine H&E specimens. Spearman’s correlation analysis and receiver-operating characteristics curves were used as statistical analyses.

**Results:**

The patient sample comprised 70 female patients (age range 32–79 years; mean age 55.4 years) with squamous cell cervical carcinoma. The interreader agreement was high ranging from intraclass coefficient (ICC) = 0.71 for entropy to ICC = 0.96 for ADCmedian. Several ADC-histogram parameters correlated strongly with the TSR. The highest correlation coefficient achieved p10 (*r* = -0.81, *p* < 0.0001). ADCmean can predict tumors with high TSR, AUC: 0.91, sensitivity: 0.91 (95% CI 0.77;0.96), specificity: 0.91 (95% CI 0.78;0.97). Several ADC-histogram parameters correlated slightly with the proliferation index Ki-67. No associations were found with TIL, E-Cadherin and vimentin. In well and moderately differentiated cancers, ADC histogram values showed stronger correlations with Ki-67 and TSR than in poorly differentiated tumors.

**Conclusion:**

ADC values are strongly associated with tumor-stroma ratio. The ADC mean can be used to predict tumors with high TSR. Associations between histopathology and ADC values depend on tumor differentiation. ADC values show only weak associations with Ki-67 and none with TIL, vimentin and E-cadherin.

## Background

Uterine cervical cancer (UCC) is the third frequent cancer and the fourth leading cause of cancer death in females worldwide [1].

Magnetic resonance imaging (MRI) is the imaging modality of choice for staging of UCC due to its excellent soft tissue contrast [2]. According to the literature, MRI can also characterize tumor microstructure in [3]. Diffusion-weighted imaging has the potential to reveal tumoral architectural details [3–5]. Previously, some studies reported that apparent diffusion coefficient (ADC) was inversely correlated with tumor cell count in UCC [4, 5].

At present, modern imaging analysis can more sensitively reflect tumor biology than conventional imaging values such as signal intensity or ADC [6, 7]. For instance, histogram analysis approach uses a distribution of voxels within a region of interest [6]. This method provides information about tumor homo-and/or heterogeneity [6]. Typical histogram parameters include percentiles, median, mode, skewness, kurtosis and entropy [6]. In UCC, it has been shown that ADC histogram parameters has been shown to discriminate between cervical physiological tissue and cancer [8]. In addition, histogram analysis can discriminate between low and high grade UCC as well UCC with and without lymph node metastases [8, 9].

According to the literature, several histopathological features such as epidermal-growth factor, hypoxia-inducible factor 1-alpha, vascular endothelial growth factor, human epidermal growth factor receptor 2 (HER 2) and histone 3 are associated with prognosis and prediction of treatment success [10–13]. Tumor-stroma ratio and tumoral lymphocytes are also associated with prognosis in UCC [14, 15]. Previously, it has been shown that ADC histogram parameters can reflect expression of epidermal-growth factor and histone 3 but not expression of vascular endothelial growth factor and hypoxia-inducible factor 1-alpha in UCC [16]. Furthermore, histogram analysis parameters of T1-weighted and T2-weighted images reflect HER2 status and EGFR expression in UCC [17].

In addition to these immunohistochemical parameters, there is ongoing research on the importance of tumor heterogeneity and the tumor stroma interaction of the tumor micro milieu [18]. There is also evidence that tumor heterogeneity plays a key role in poor oncologic outcomes [19].

These previous studies led to the assumption that imaging could non-invasively predict certain aspects of the underlying tumor microstructure and also the malignant potential.

In addition to the histogram analysis mentioned above, other imaging analyses have been used to reflect tumor heterogeneity with FDG-PET volumetry [20]. Very promising results have been demonstrated for radiomics analyses a more comprehensive approach to assess tumor heterogeneity with promising results for diagnostic purposes and treatment prediction [21, 22].

However, further studies with larger patient samples and more complex pathological analyses are warranted to elucidate the comprehensive relationships between imaging and histopathology. This is especially true, considering that imaging is the only modality that can determine the tumor heterogeneity as a whole, whereas histopathology can only assess one portion of the tumor.

Therefore, the purpose of the present study was to investigate possible relationships between ADC histogram parameters and histopathological features including the expression of Ki-67, tumor-stroma ratio and tumoral infiltrating immune cells in UCC.

## Methods

### Patient acquisition

All consecutive patients with UCC at our tertiary referral hospital were retrospectively assessed. The study was performed after approval of the local Ethics commission in accordance with the ethical standards of the institutional and/or national research committee and with the 1964 Helsinki declaration and its later amendments or comparable ethical standards (Ethical code: 012/13–28,012,013).

Inclusion criteria for the present study were biopsy-proven squamous cell UCC. MRI had to be performed before the biopsy and must include a DWI sequence. The tumor had to be visible on the ADC maps. Exclusion criteria were available DWI sequence, severe artifacts of the ADC maps, no visible tumor on the ADC map, and lack of histopathology specimens.

All patients were classified according to the “Fédération Internationale de Gynécologie et d’Obstétrique” classification [23].

The recruitment period was from 2015–2023. Prior to 2018, the DWI sequence was not routinely included in the MRI protocol.

**Fig. 1:**
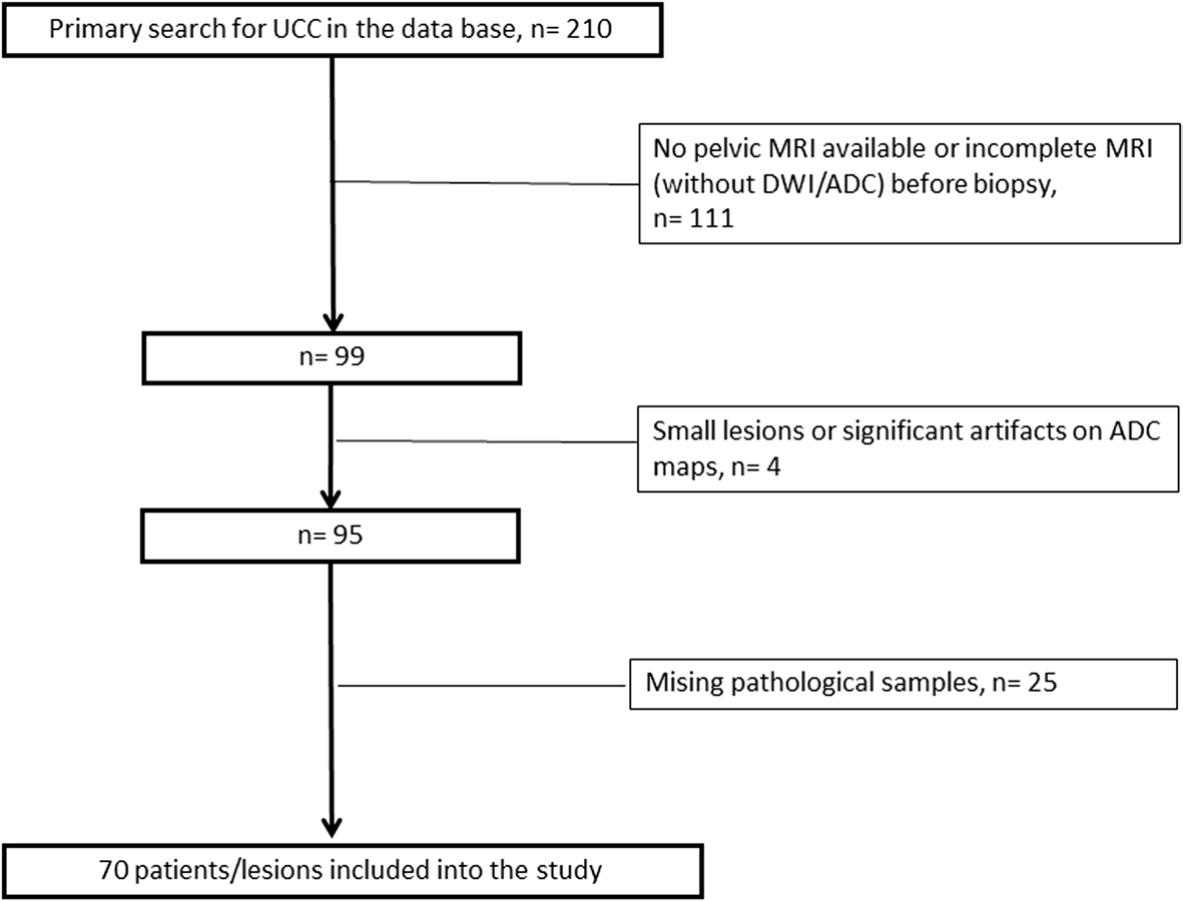
Study flow chart of the acquisition of the patient sample. The final patient sample was comprised of 70 patients

The primary search comprised 210 patients. Thereof, 111 patients were excluded due to missing DWI sequence of the MRI. Then, 4 patients were excluded due to artifacts or not visible tumor on MRI. Finally, 25 patients had no corresponding histopathology specimens. Figure 1 provides the study flow chart of the patient sample. All patients were investigated by MRI before any form of treatment and the biopsy procedure.

### MRI

In all cases, pelvic MRI was performed with a 1.5 T scanner (Aera, Siemens, Erlangen, Germany. Our investigation protocol included the following sequences: an axial T2 weighted (T2w) turbo spin echo (TSE) sequence (TR/TE: 5590/105), a sagittal T2w TSE sequence (TR/TE: 4110/131), an axial T1 weighted (T1w) TSE sequence (TR/TE:1310/12), an axial T1 TSE sequence after intravenous application of contrast medium (0.1 mmol/kg body weight Gadobutrol, Bayer Healthcare, Germany) (TR/TE:912/12), and a sagittal post contrast T1 TSE (TR/TE: 593/12). DWI was performed using a multi-shot SE-EPI sequence (b 0 and b 1000 s/mm2, repetition time: 4900 ms; echo time: 105 ms; slice thickness: 5 mm; matrix: 88 × 134; field of view: 450 × 450 mm.

### Histogram analysis of ADC values

**Fig. 2:**
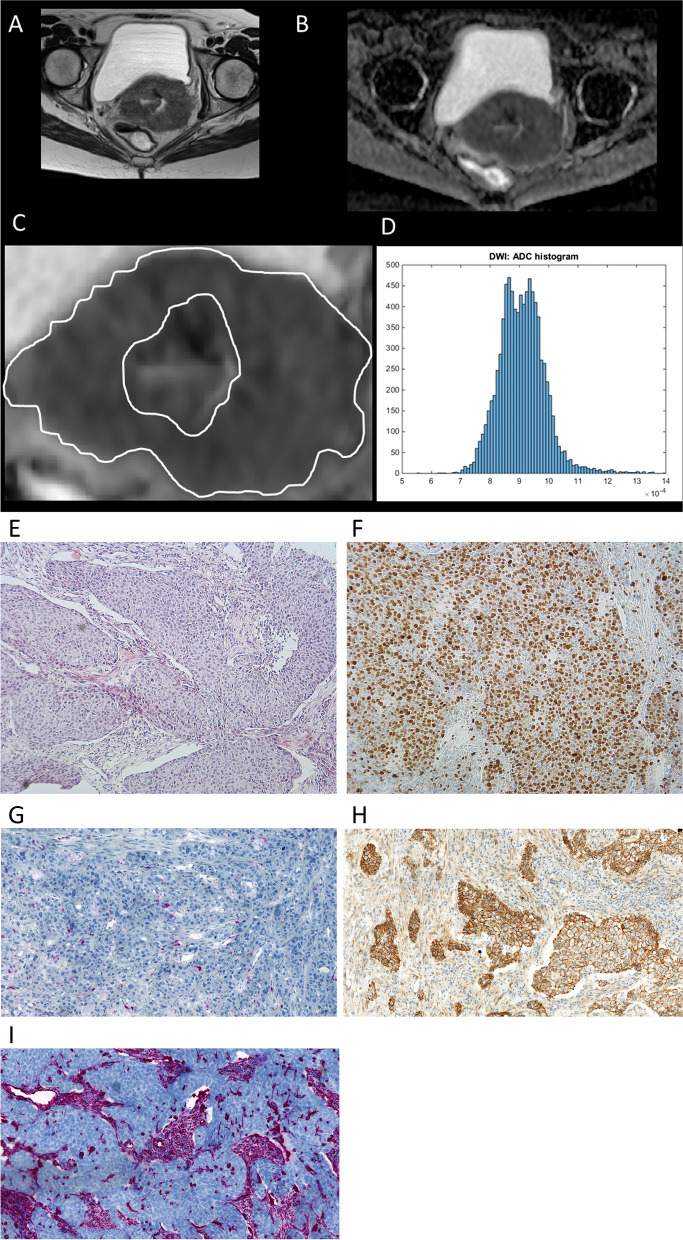
Representative patient of the present cohort. **A** T2-weighted axial image showing the large, inhomogeneous tumor. **B** ADC-map of the patient. **C** Drawn region of interest within the boundaries of the tumor. **D** The resulting ADC histogram for this patient. It shows a high kurtosis and a low skewness. Histopathological findings of the tumor shows a low stromal area (E, H&E staining), a high proliferation index (**F**, MIB staining), low number of CD 45 positive cells (**G**, CD 45 staining), a high expression of E-cadherin (**H**, E-cadherin staining), and a moderate expression of vimentin (**I**, vimentin staining)

Automatically generated ADC maps were further processed offline with a custom-made Matlab-based application (The Mathworks, Natick, MA). The ADC maps were displayed within a graphical user interface (GUI), which enables the reader to scroll through the slices and draw a volume of interest (VOI) at the tumor’s boundary, in accordance to the T2-weighted images (whole lesion measure). All measures were performed by one author independently to each other and blinded to histopathology (AS with 19 years of experience in radiology). The ROIs were modified in the GUI and saved (in Matlab-specific format) for later processing. Following parameters were calculated: mean (ADCmean), maximum (ADCmax), minimum (ADCmin), median, 10th (p10 ADC), 25th (p25 ADC), 75th (p75 ADC), 90th (p90 ADC) percentile, and mode (ADC mode). Additionally, histogram parameters were calculated comprising kurtosis, skewness and entropy. For interreader agreement a subset of randomly selected 20 patients were measured by a second reader with 8 years of general radiology experience. Figure 2 provides a representative case of the present cohort to demonstrate the measurement.

### Histopathological analysis

In every patient, the biopsy specimens before the MRI was further analyzed. Histopathology evaluation was performed by one experienced pathologist (AKH) without knowledge of the patients or imaging data. Formalin-fixed, paraffin-embedded tissue serial Sects. (2 µm) were dewaxed in xylol and rehydrated by descending concentrations of ethanol. For each specimens, standard hematoxylin and eosin (HE) staining and immunohistochemistry were performed. For antigen detection, we used the automated immunohistochemistry slide staining system VENTANA BenchMark ULTRA (Roche Diagnostics GmbH), the VENTANA iVIEW DAB Detection Kit (Roche Diagnostics GmbH), and the indirect biotin-streptavidin method before counterstaining with Haemalaun solution. Antigen retrieval was performed with CC1mild, followed by incubation with specific primary antibodies recognizing CD45/leucocyte common antigen (polyclonal mouse antibody, clone 2B11 + PD7/26; DAKO/ Agilent #M0701) or Ki-67 (polyclonal mouse antibody, clone Mib1; DAKO/Agilent #M7240), at 36 °C for 32 min, dilution 1:500 or 1:100, respectively. Every histopathological parameter was evaluated in five power fields (× 40; 0.23 mm^2^ per field). For each specimen, the mean values of the quantified parameter were calculated. Tumor-stroma ratio (TSR) was evaluated on the HE-stained specimens and percentages were given per tumor and stroma content separately. Density of tumor-infiltrating immune cells were estimated as a mean of overall cell counts or CD45 + leucocytes per high power field, respectively. The extracellular matrix was stained by vimentin (DAKO, clone Vim 3B4, dilution 1:200) and the percentage of the stained HPF was measured. The mouse anti-E-cadherin monoclonal primary antibody (Clone: NCH-38; M3612; DakoCytomation, Denmark) was used to stain for E-cadherin. The rate of proliferation was indicated by the percentage of Ki-67-positive cells from all tumor cells (Ki-67-index). Ki-67 was defined as the highest value of the five measurements. Histopathological evaluation was done with the Nikon ECLIPSE Ni-E microscope. Figure 2 (images E-I) shows a representative case of the sample.

Subgroup analyses were performed stratified to the tumor grading. Well and moderately differentiated tumors were grouped together and compared with poorly differentiated tumors.

### Statistical analysis

The statistical analysis and graphics creation were performed using GraphPad Prism 10 (GraphPad Software, La Jolla, CA, USA) and SPSS (IBM, Version 25.0; Armonk, NY, USA). Collected data were evaluated by means of descriptive statistics (absolute and relative frequencies). Spearman’s correlation coefficient (r) was used to analyze associations between the investigated ADC-histogram parameters and the investigated histopathological parameters after testing for normality distribution. Group differences were calculated with Mann–Whitney-U-test. Interreader agreement was investigated by means of intraclass-coefficient (ICC). Diagnostic accuracy was tested with receiver operating characteristics curve (ROC) with area under the curve (AUC). Results are presented with 95% confidence intervals (CI). In all instances, *p-v*alues < 0.05 were used to demonstrate statistical significance.

## Results

The patient sample consisted of 70 female patients (age range 32–79 years; mean age 55.4 years) with squamous cell cervical carcinoma. Table 1 summarizes the characteristics of the patient sample.

**Table 1:** Overview of the investigated patient sample

**FIGO stage**	
** IB1**	24 (34.2%)
** IB2**	5 (7.1%)
** IIA**	5 (7.1%)
** IIB**	23 (32.9%)
** IIIA**	3 (4.3%)
** IIIB**	8 (11.5%)
** IVA**	2 (2.9%)
**T stage**	
1	20 (29%)
2	31 (44%)
3	14 (20%)
4	5 (7%)
**N stage**	
N0	27 (39%)
N +	43 (61%)
**M stage**	
M0	57 (%)
M1	13 (%)
**Tumor grade**	
G1	3 (4%)
G2	33 (47%)
G3	34 (49%)

An overview of the ADC-histogram parameters and the histopathological parameters are given by Tables 2 and 3. The interreader agreement was high ranging from ICC = 0.71 for entropy to ICC = 0.96 for ADCmedian.

**Table 2:** Descriptive overview of the investigated ADC-histogram parameters

ADC values	M ± SD
ADC Mean	0.51 ± 0.43
ADC Min	0.34 ± 0.31
ADC Max	0.76 ± 0.65
ADC P10	0.43 ± 0.37
ADC P25	0.46 ± 0.39
ADC Median	0.50 ± 0.43
ADC P75	0.54 ± 0.46
ADC P90	0.59 ± 0.50
ADC Mode	0.49 ± 0.41
Kurtosis	4.64 ± 3.24
Skewness	0.63 ± 0.84
Entropy	3.71 ± 0.58

**Table 3:** Descriptive overview of the investigated histopathological features of the tumors

	**M ± SD**
Tumor-stroma ratio	31.1 ± 19.4
Ki-67, %	68.5 ± 15.6
Expression of E-cadherin	3.6 ± 0.7
Expression of Vimentin	4.8 ± 14.9
Stromal TIL	41.0 ± 24.5
Tumoral TIL	3.3 ± 3.4

### Correlation analysis

The results of correlation analysis are given by Tables 4, 5 and 6.

**Table 4:** Spearman’s correlation analysis between the ADC histogram parameters and the investigated histopathological features

ADC histogram parameters	Ki-67	Tumor-stroma-ratio	E-Cadherin	Vimentin	Stromal TIL	Tumoral TIL
mean	***r* = −0.30, *p* = 0.01**	***r* = −0.80, *p* < 0.0001**	*r* = −0.04, *p* = 0.72	*r* = 0.01, *p* = 0.87	*r* = −0.07, *p* = 0.55	*r* = −0.14, *p* = 0.25
Min	***r* = −0.30, *p* = 0.01**	***r* = −0.75, *p* < 0.0001**	*r* = −0.04, *p* = 0.72	*r* = −0.04, *p* = 0.73	*r* = 0.03, *p* = 0.77	*r* = −0.09, *p* = 0.43
Max	***r* = −0.28, *p* = 0.01**	***r* = −0.71, *p* < 0.0001**	*r* = −0.10, *p* = 0.37	*r* = −0.01, *p* = 0.91	*r* = −0.05, *p* = 0.63	*r* = −0.12, *p* = 0.29
P10	***r* = −0.31, *p* = 0.008**	***r* = −0.81, *p* < 0.0001**	*r* = −0.05, *p* = 0.65	*r* = 0.04, *p* = 0.72	*r* = −0.05, *p* = 0.66	*r* = −0.14, *p* = 0.24
P25	***r* = −0.30, *p* = 0.01**	***r* = −0.79, *p* < 0.0001**	*r* = −0.05, *p* = 0.65	*r* = 0.04, *p* = 0.70	*r* = −0.06, *p* = 0.57	*r* = −0.14, *p* = 0.24
P75	***r* = −0.30, *p* = 0.01**	***r* = −0.78, *p* < 0.0001**	*r* = −0.04, *p* = 0.75	*r* = 0.01, *p* = 0.89	*r* = −0.06, *p* = 0.57	*r* = −0.14, *p* = 0.26
P90	***r* = −0.29, *p* = 0.01**	***r* = −0.80, *p* < 0.0001**	*r* = −0.04, *p* = 0.77	*r* = −0.005, *p* = 0.96	*r* = −0.07, *p* = 0.52	*r* = −0.14, *p* = 0.25
Median	***r* = −0.27, *p* = 0.02**	***r* = −0.80, *p* < 0.0001**	*r* = −0.04, *p* = 0.75	*r* = 0.03, *p* = 0.77	*r* = −0.06, *p* = 0.58	*r* = −0.14, *p* = 0.23
Mode	*r* = −0.31, *p* = 0.08	***r* = −0.80, *p* < 0.0001**	*r* = −0.07, *p* = 0.54	*r* = 0.05, *p* = 0.63	*r* = −0.05, *p* = 0.67	*r* = 0.13, *p* = 0.30
Kurtosis	*r* = −0.19, *p* = 0.11	*r* = 0.003, *p* = 0.97	*r* = −0.22, *p* = 0.06	*r* = 0.002, *p* = 0.98	*r* = 0.06, *p* = 0.58	*r* = −0.06, *p* = 0.61
Skewness	*r* = −0.11, *p* = 0.36	*r* = 0.09, *p* = 0.44	*r* = −0.20, *p* = 0.09	*r* = −0.20, *p* = 0.08	*r* = 0.03, *p* = 0.77	*r* = −0.06, *p* = 0.61
Entropy	*r* = −0.09, *p* = 0.44	*r* = 0.14, *p* = 0.24	*r* = −0.08, *p* = 0.50	*r* = −0.04, *p* = 0.69	*r* = 0.02, *p* = 0.81	*r* = −0.08, *p* = 0.49

Statistically significant correlations are highlighted in bold

**Table 5:** Spearman’s correlation analysis in the subgroup analysis of the well and moderate differentiated tumors (G1 + 2)

ADC histogram parameters	Ki-67	Tumor-stroma-ratio	E- Cadherin	Vimentin	Stromal TIL	Tumoral TIL
mean	***r* = -0.49, *p* = 0.002**	***r* = -0.88, *p* < 0.0001**	*r* = -0.13, *p* = 0.44	*r* = -0.08, *p* = 0.63	*r* = -0.25, *p* = 0.14	*r* = -0.08, *p* = 0.62
Min	***r* = -0.46, *p* = 0.004**	***r* = -0.80, *p* < 0.0001**	*r* = 0.08, *p* = 0.63	*r* = -0.08, *p* = 0.63	*r* = 0.09, *p* = 0.57	*r* = -0.02, *p* = 0.89
Max	***r* = -0.36, *p* = 0.02**	***r* = -0.76, *p* < 0.0001**	*r* = 0.06, *p* = 0.71	*r* = -0.04, *p* = 0.77	*r* = -0.17, *p* = 0.30	*r* = -0.03, *p* = 0.83
P10	***r* = -0.46, *p* = 0.004**	***r* = -0.91, *p* < 0.0001**	*r* = 0.06, *p* = 0.68	*r* = 0.04, *p* = 0.72	*r* = -0.22, *p* = 0.19	*r* = -0.06, *p* = 0.73
P25	***r* = -0.36, *p* = 0.02**	***r* = -0.91, *p* < 0.0001**	*r* = 0.09, *p* = 0.58	*r* = 0.09, *p* = 0.57	*r* = -0.26, *p* = 0.13	*r* = -0.07, *p* = 0.65
P75	***r* = -0.46, *p* = 0.004**	***r* = -0.87, *p* < 0.0001**	*r* = 0.15, *p* = 0.37	*r* = 0.12, *p* = 0.46	*r* = -0.25, *p* = 0.14	*r* = -0.08, *p* = 0.64
P90	***r* = -0.45, *p* = 0.005**	***r* = -0.84, *p* < 0.0001**	*r* = 0.16, *p* = 0.33	*r* = 0.03, *p* = 0.84	*r* = -0.24, *p* = 0.16	*r* = -0.06, *p* = 0.70
Median	***r* = -0.48, *p* = 0.002**	***r* = -0.90, *p* < 0.0001**	*r* = 0.14, *p* = 0.38	*r* = 0.12, *p* = 0.47	*r* = -0.26, *p* = 0.11	*r* = -0.06, *p* = 0.57
Mode	***r* = -0.44, *p* = 0.006**	***r* = -0.91, *p* < 0.0001**	*r* = 0.10, *p* = 0.54	*r* = 0.16, *p* = 0.33	*r* = -0.25, *p* = 0.14	*r* = -0.05, *p* = 0.74
Kurtosis	*r* = -0.11, *p* = 0.49	*r* = -0.11, *p* = 0.50	***r* = -0.35, *p* = 0.03**	*r* = -0.10, *p* = 0.53	*r* = 0.28, *p* = 0.09	*r* = 0.09, *p* = 0.56
Skewness	*r* = 0.08, *p* = 0.96	*r* = 0.10, *p* = 0.52	*r* = -0.17, *p* = 0–32	***r* = -0.41, *p* = 0.01**	*r* = 0.21, *p* = 0.22	*r* = -0.07, *p* = 0.67
Entropy	*r* = 0.21, *p* = 0.20	*r* = 0.22, *p* = 0.19	*r* = -0.21, *p* = 0.21	*r* = 0.02, *p* = 0.87	*r* = 0.12, *p* = 0.46	*r* = 0.15, *p* = 0.38

Statistically significant correlations are highlighted in bold

**Table 6:** Spearman’s correlation analysis in the subgroup analysis of poorly differentiated tumors (G3)

ADC histogram parameters	Ki-67	Tumor-stroma-ratio	E -Cadherin	Vimentin	Stromal TIL	Tumoral TIL
mean	*r* = -0.16, *p* = 0.34	***r* = -0.73, *p* < 0.0001**	*r* = -0.21, *p* = 0.22	*r* = -0.04, *p* = 0.80	*r* = 0.11, *p* = 0.50	*r* = -0.20, *p* = 0.25
Min	*r* = -0.15, *p* = 0.36	***r* = -0.70, *p* < 0.0001**	*r* = -0.19, *p* = 0.26	*r* = -0.004, *p* = 0.98	*r* = 0.19, *p* = 0.26	*r* = -0.15, *p* = 0.39
Max	*r* = -0.18, *p* = 0.30	***r* = -0.68, *p* < 0.0001**	*r* = -0.32, *p* = 0.06	*r* = 0.006, *p* = 0.97	*r* = 0.10, *p* = 0.55	*r* = -0.19, *p* = 0.26
P10	*r* = -0.18, *p* = 0.28	***r* = -0.72, *p* < 0.0001**	*r* = -0.18, *p* = 0.30	*r* = -0.02, *p* = 0.89	*r* = 0.12, *p* = 0.47	*r* = -0.19, *p* = 0.26
P25	*r* = -0.18, *p* = 0.28	***r* = -0.71, *p* < 0.0001**	*r* = -0.20, *p* = 0.24	*r* = -0.02, *p* = 0.87	*r* = 0.12, *p* = 0.49	*r* = -0.21, *p* = 0.24
P75	*r* = -0.16, *p* = 0.36	***r* = -0.72, *p* < 0.0001**	*r* = -0.22, *p* = 0.19	*r* = -0.04, *p* = 0.78	*r* = 0.11, *p* = 0.51	*r* = -0.19, *p* = 0.28
P90	*r* = -0.12, *p* = 0.47	***r* = -0.74, *p* < 0.0001**	*r* = -0.25, *p* = 0.15	*r* = -0.04, *p* = 0.81	*r* = 0.12, *p* = 0.48	*r* = -0.18, *p* = 0.30
Median	*r* = -0.17, *p* = 0.32	***r* = -0.72, *p* < 0.0001**	*r* = -0.21, *p* = 0.22	*r* = -0.04, *p* = 0.78	*r* = 0.12, *p* = 0.48	*r* = -0.20, *p* = 0.25
Mode	*r* = -0.19, *p* = 0.27	***r* = -0.72, *p* < 0.0001**	*r* = -0.24, *p* = 0.16	*r* = -0.02, *p* = 0.90	*r* = 0.12, *p* = 0.47	*r* = -0.18, *p* = 0.29
Kurtosis	*r* = -0.15, *p* = 0.38	*r* = 0.14, *p* = 0.40	*r* = -0.08, *p* = 0.61	*r* = 0.10, *p* = 0.54	*r* = -0.10, *p* = 0.55	*r* = 0.01, *p* = 0.92
Skewness	*r* = -0.21, *p* = 0.23	*r* = 0.05, *p* = 0.76	*r* = -0.30, *p* = 0.08	*r* = -0.005, *p* = 0.97	*r* = -0.12, *p* = 0.48	*r* = -0.06, *p* = 0.71
Entropy	*r* = -0.21, *p* = 0.21	*r* = 0.04, *p* = 0.80	*r* = 0.13, *p* = 0.43	*r* = -0.14, *p* = 0.43	*r* = -0.02, *p* = 0.89	*r* = -0.29, *p* = 0.09

Statistically significant correlations are highlighted in bold

**Fig. 3:**
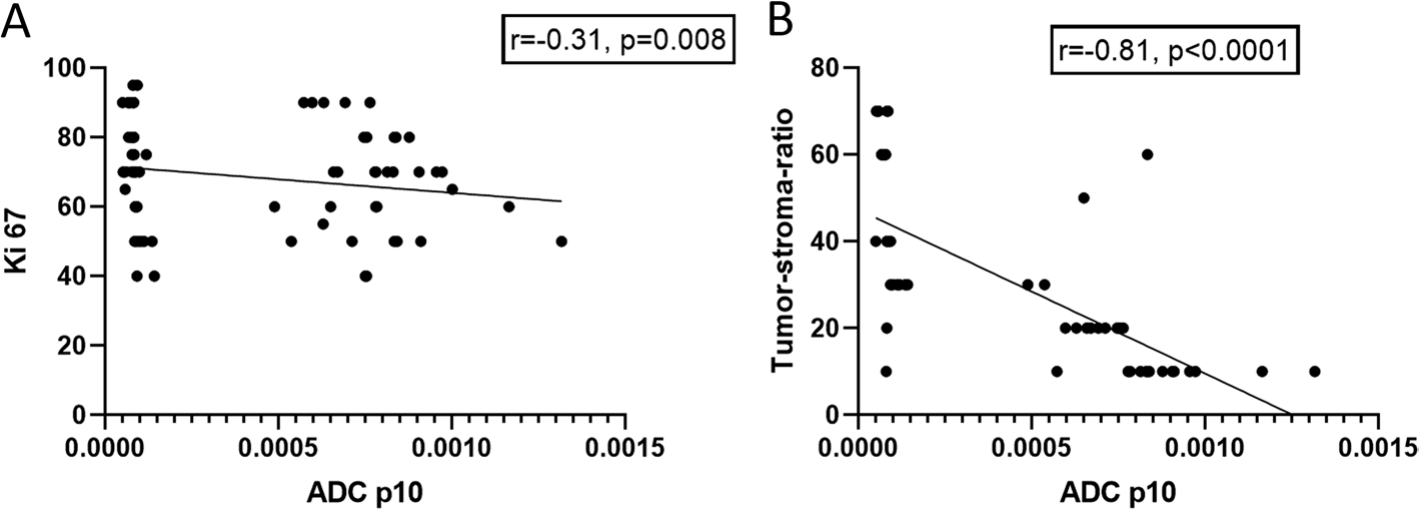
**A** Spearman’s correlation analysis between ADC p10 with the Ki-67 index, *r* = −0.31, *p* = 0.008. B Spearman’s correlation analysis between ADC p10 with the tumor-stroma ratio, *r* = −0.81, *p* < 0.0001

ADC-histogram parameters correlated weakly with the proliferation index Ki-67. The strongest correlation coefficient was identified for p10 (*r* = −0.31, *p* = 0.008) (Fig. 3a).

Several ADC-histogram parameters correlated strongly with tumor-stroma ratio, the highest correlation coefficient achieved p10 (*r* = −0.81, *p* < 0.0001) (Fig. 3b).

There were no correlations between ADC values and TIL, E-Cadherin and vimentin.

At the next step, a subanalysis of associations between ADC histogram values and histopathology was performed in well and moderate differentiated tumors separately.

In well and moderately differentiated cancers, ADC histogram values showed stronger correlations with Ki-67 and tumor-stroma ratio than in poorly differentiated tumors (Table).

**Fig. 4:**
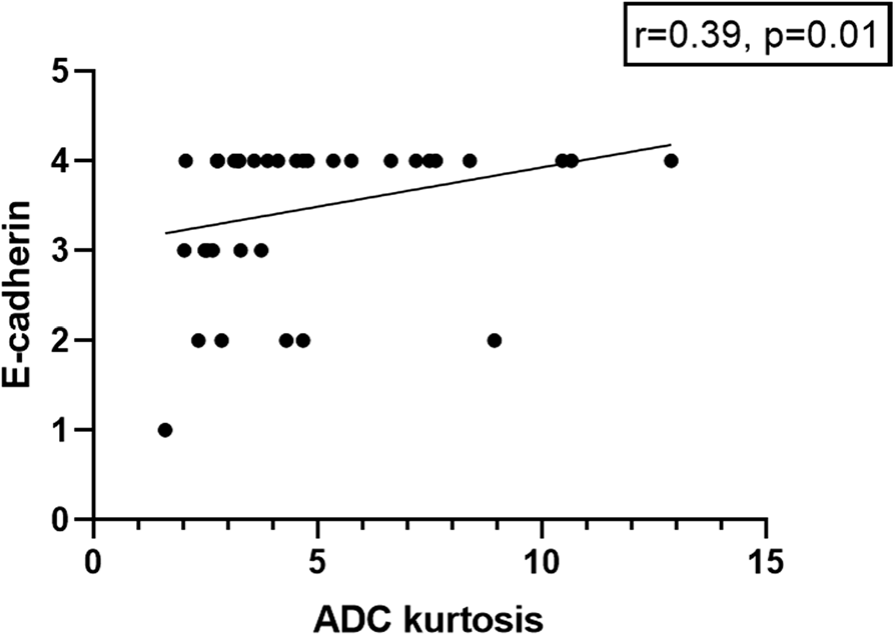
Subgroup analysis of the well and moderate differentiated tumors. Spearman’s correlation analysis between kurtosis and E-cadherin expression (*r* = −0.35, *p* = 0.03)

Furthermore, skewness correlated with vimentin expression (*r* = −0.41, *p* = 0.01) as well as between kurtosis and E-cadherin expression (*r* = −0.35, *p* = 0.03) (Fig. 4).

### Prediction of histopathological features

The prediction for ADC-histogram parameters for tumors with high tumor-stroma ratio defined by the median value of 30 was carried out using ROC analysis.

**Fig. 5:**
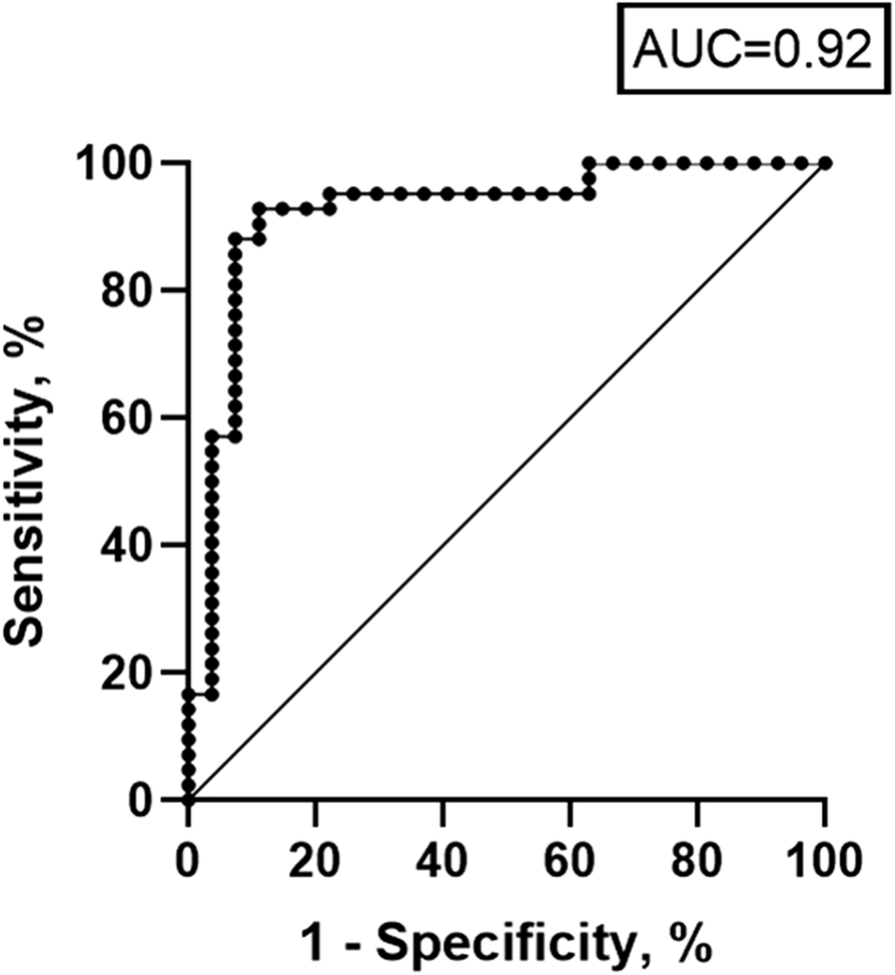
Receiver operating characteristics curve for the prediction of high tumor-stroma ratio tumors using ADCmean. The resulting AUC is 0.91 (95%CI 0.84;0.98) with a sensitivity of 0.91 (95%CI 0.77;0.96) and a specificity of 0.91 (95%CI 0.78;0.97)

The highest AUC achieved ADCmean: sensitivity of 0.91 (95% CI 0.77;0.96) and a specificity of 0.91 (95% CI 0.78;0.97). The corresponding ROC curve is shown in Fig. 5.

## Discussion

The present study elucidated the complex interactions between ADC histogram parameters and histopathologic features in UCC. A strong inverse association was found between ADC values and the tumor-stroma ratio, whereas only a weak association was demonstrated with the proliferation potential.

No correlations were found for TIL, vimentin, and E-cadherin expression in the overall cohort. However, in well and moderately differentiated tumors, there were associations with vimentin and E-cadherin. Moreover, the associations with proliferation potential and tumor-stroma ratio were stronger in the well and moderate differentiated tumor group. This is a key finding of the present study that the associations between ADC values and histopathology features are dependent on the tumor grading.

These results are in good agreement with previous investigation, which could also show a similar trend, for example in head and neck cancer [24].

The present study provides new insights into the complex interactions between diffusion within tumor tissue quantified by MRI and the underlying microstructural processes.

This is especially of great importance as the interactions between tumor, infiltrating immune cells and extracellular matrix, termed microenvironment is of great prognostic importance [25–28]. One important feature is the remodeling of peritumoral stroma determined by a switch from fibroblasts to myofibroblasts, extracellular matrix alterations (known as desmoplastic change), and neo-angiogenesis [27]. The presence of desmoplastic changes is high in UCC with 80.6% of cases and leads to an inferior overall survival (hazard ratio 3.8 [95% CI 1.4–10.4], *p* = 0.002) [27].

As these microstructural changes within the tumors also imply prognostic relevance, it could be crucial to implement ADC histogram parameters as a way to non-invasively characterize the whole tumor. Beyond that, contrary to the biopsy specimens, which can only provide insight into a small area of the tumor, the MRI could provide insight of the whole tumor.

There is a no doubt that ADC values are directly and inversely correlated with the cell density, as was demonstrated in various tumor entities [28]. However, there are distinctive differences between the different tumors, ranging from weak to strong [28]. Moreover, there was a clear trend for ADC values to correlate inversely with the Ki-67 index [4, 29].

For cervical cancer, a preliminary explorative study could not demonstrate an association with cell density or Ki-67 index in UCC [9]. Yet, there was an inverse correlation with p53 expression and ADCentropy [9]. Another study could show a moderate inverse correlation between ADCmin and Ki-67 index *r* = −0.48, *p* = 0.03 [4].

In another study, the associations between ADC histogram analysis and immunohistochemical stainings of neoangiogenesis, epidermal-growth factor expression and histone 3 expression were explored [16]. There were no associations with the neoangiogensis related factors, but with epidermal-growth factor and histone 3 expression. The highest correlation was observed for ADCp75 (*p* = −0.562, *P* = 0.015) and ADCp25 with histone 3 (*p* = −0.610, *P* = 0.007) [16].

The role of tumor infiltrating lymphocytes and the tumor-stroma ratio is of utter importance in UCC [14, 15]. The main finding of the present study is the ability of ADC values to predict tumors with high tumor-stroma ratio but it is not able to reflect the amount of infiltrating lymphocytes. It seems plausible that the microstructural characteristics of a small subset of cells within the tumor cannot be reflected by a whole tumor ADC measurement. Yet, in a similar study investigating head and neck cancer, there was a slight trend for ADC entropy to correlate with the tumor infiltrating lymphocytes of the tumor compartment [30].

One interesting aspect of the present study is that there was a statistical signal for ADCkurtosis to reflect the E-cadherin expression and ADCskewness to correlate with vimentin expression in the well and moderate differentiated tumors. There is definite need for the characterization between poor and well differentiated UCC. Moreover, it is well established that ADC histogram parameters differ significantly between these groups [8].

An important aspect to discuss is the size of the tumor. The tumors studies in the present cohort were all visible on DWI and the ADC maps. There is evidence that DWI volume and T2-weighted assessed tumor volume can be considered similar [31, 32]. However, there may be very small tumors, that are not visible on DWI and therefore cannot be further assessed with ADC histogram analysis, especially in FIGO IA [23].

Another issue is the tumor heterogeneity induced by large tumors with central necrosis [20]. In these cases, the spatial differences between biopsy and the histopathological evaluation and the ADC histogram analysis of the MRI might lead to a worse correlation between imaging and histopathology. This could be a reason for some of the non-existing correlations in the present study.

Beyond that, this could be one reason for the differences identified between the subgroup analysis accordingly to the tumor grading. The weaker correlations observed in the poorly differentiated tumor group may be due to the higher heterogeneity of the tumors and the higher risk of biopsy sampling bias. There is clear need to further elucidate the influence of tumor grading on immunohistochemical and imaging parameters.

The present study is not free from limitations. First, it is a retrospective study with known inherent bias. However, the imaging and pathologic analysis were performed independently and blinded to each other to reduce possible bias. It should be considered that there may be spatial inconsistencies between imaging and histopathology, which may have an influence on the correlations between the two modalities. Second, the patient sample is comprised from a single center with possible selection bias. It could reduce the external validity of the present results. Third, the study only included patients with squamous cell carcinomas, which need to be considered. There may be no translation of the present results to the rare cervical adenocarcinomas. Fourth, the study only included a small set of immunohistochemical parameters. It would be interesting to further analyse more parameters, such as programmed-death ligand 1, HER2 or even mutations [33].

## Conclusions

ADC values are strong associated with tumor-stroma ratio in uterine cervical cancer and can be used to predict tumors with a high stroma in a non-invasive manner. However, ADC parameters are not able to reflect tumor-infiltrating lymphocytes. Associations between ADC histogram parameters and histopathology depend on tumor grading. ADC values correlate stronger with proliferation potential and tumor-stroma ratio in well and moderate differentiated tumors than in poorly differentiated cancers.

## Data Availability

No datasets were generated or analysed during the current study.

## Abbreviations

ADC: Apparent diffusion coefficient
AUC: Area under the curve
CI: Confidence interval
DWI: Diffusion-weighed imaging
ICC: Intraclass coefficient
MRI: Magnetic resonance imaging
ROC: Receiver operating characteristics
UCC: Uterine cervical cancer
TIL: Tumor infiltrating lymphocyte
